# Type 2 diabetes patients’ views on prevention of hypoglycaemia – a mixed methods study investigating self-management issues and self-identified causes of hypoglycaemia

**DOI:** 10.1186/s12875-021-01466-0

**Published:** 2021-06-14

**Authors:** Stijn Crutzen, Tessa van den Born-Bondt, Petra Denig, Katja Taxis

**Affiliations:** 1 Department of Clinical Pharmacy and Pharmacology, University Medical Centre Groningen, University of Groningen, Groningen, Netherlands; 2 Unit of PharmacoTherapy, Epidemiology and Economics, Groningen Research Institute of Pharmacy, University of Groningen, Groningen, The Netherlands

**Keywords:** Type 2 diabetes, Hypoglycaemia, Self-management, Patient perspective, Mixed methods

## Abstract

**Background:**

Hypoglycaemia is a common and potentially avoidable adverse event in people with type 2 diabetes (T2D). It can reduce quality of life, increase healthcare costs, and reduce treatment success. We investigated self-management issues associated with hypoglycaemia and self-identified causes of hypoglycaemia in these patients.

**Methods:**

In this mixed methods study qualitative semi-structured interviews were performed, which informed a subsequent quantitative survey in T2D patients. All interviews were audio recorded, transcribed verbatim and coded independently by two coders using directed content analysis, guided by the Theoretical Domains Framework. Descriptive statistics were used to quantify the self-management issues and causes of hypoglycaemia collected in the survey for the respondents that had experienced at least one hypoglycaemic event in the past.

**Results:**

Sixteen participants were interviewed, aged 59–84 years. Participants perceived difficulties in managing deviations from routine, and they sometimes lacked procedural knowledge to adjust medication, nutrition or physical activity to manage their glucose levels. Grief and loss of support due to the loss of a partner interfered with self-management and lead to hypoglycaemic events. Work ethic lead some participant to overexerting themselves, which in turn lead to hypoglycaemic events. The participants had difficulties preventing hypoglycaemic events, because they did not know the cause, suffered from impaired hypoglycaemia awareness and/or did not want to regularly measure their blood glucose. When they did recognise a cause, they identified issues with nutrition, physical activity, stress or medication. In total, 40% of respondents reported regular stress as an issue, 24% reported that they regularly overestimated their physical abilities, and 22% indicated they did not always know how to adjust their medication. Around 16% of patients could not always remember whether they took their medication, and 42% always took their medication at regular times. Among the 83 respondents with at least one hypoglycaemic event, common causes for hypoglycaemia mentioned were related to physical activity (67%), low food intake (52%), deviations from routine (35%) and emotional burden (28%). Accidental overuse of medication was reported by 10%.

**Conclusion:**

People with T2D experience various issues with self-managing their glucose levels. This study underlines the importance of daily routine and being able to adjust medication in relation to more physical activity or less food intake as well as the ability to reduce and manage stress to prevent hypoglycaemic events.

**Supplementary Information:**

The online version contains supplementary material available at 10.1186/s12875-021-01466-0.

## Introduction

Hypoglycaemia is a common and potentially avoidable adverse event of treatment with insulin or medication which stimulates secretion of insulin in people with type 2 diabetes (T2D). Hypoglycaemia can reduce quality of life, increase healthcare costs, and reduce treatment success of glucose lowering medication [1–3]. Severe cases of hypoglycaemia can lead to hospitalization, brain dysfunction and increased mortality [4–7]. The reported rates of hypoglycaemia in people with T2D vary widely, depending on the study population, study design and severity of the hypoglycaemia studied [8]. In a four week prospective global study in people with T2D, 47% of participants using insulin reported at least one event and 9% reported at least one severe event [9]. In a Dutch study in people with T2D, 41% of participants using insulin reported at least one event and 4% reported at least one severe event in the past year [10]. However studies with continues glucose measurement indicate that hypoglycaemia is frequently unrecognized and more common than previously believed [11].

The causes of hypoglycaemia are multifactorial and include the intrinsic risks of specific medication and comorbidities. Behavioural factors of medication use, physical activity and nutrition also influence the occurrence of hypoglycaemic events [12–16]. Few studies have looked at possible causes for hypoglycaemia from the perspective of patients. In those studies, participants reported that they experienced hypoglycaemic events due to delayed or skipped meals, alcohol use, dieting and inconsistent eating patterns [12, 13, 15, 16]. Also, incorrect timing or dosing of insulin, stress or exercising more or more vigorously than planned were reported as possible causes of hypoglycaemia [12, 13, 15, 16]. These studies, however, mostly used questionnaires with predefined answers, which limits the possible range of causes that can be identified. One study used a qualitative design, where patients were not restricted in their reporting, but this study focussed only on the impact of fasting on self-management of glucose levels [17].

Self-management can be challenging for people with T2D. Social support from family members and other personal networks as well as support from health care providers can improve self-management of T2D [18, 19]. For chronic diseases, self-management is the ability of people to manage their disease in order to reduce the negative impact on their physical and psychosocial wellbeing [20]. This often requires lifestyle changes, monitoring of the disease and adequate medication taking behaviour. For people with T2D these requirements change over the course of their disease [21]. When diagnosed with T2D, self-management and self-management support is mostly focussed on lifestyle changes, such as increasing physical activity and improving diet [22]. As the disease progresses and medication is added, self-monitoring of blood glucose and adjusting medication accordingly may become necessary.

Many studies investigated the risk factors for hypoglycaemia from a clinical perspective, but in-depth information on the self-management issues and behavioural factors that contribute to hypoglycaemia from the patient perspective is lacking [12–16]. Our first aim was therefore to explore self-management issues associated with hypoglycaemia and subsequently quantify these issues in a larger population. Among patients who have experienced at least one hypoglycaemic event, we aimed to explore and quantify the factors these patients identify as the causes of the hypoglycaemia.

## Subjects, materials and methods

### Design

We used a mixed methods study design, combining in-depth semi-structured interviews and a cross-sectional survey. The interviews were intended to provide in-depth information from the patient perspective. In addition, the results were used for the development of a survey to quantify self-management issues and causes of hypoglycaemia. The Theoretical Domains Framework (TDF) was used as a framework to structure the topics of the interviews and identify domains potentially related to changes in behaviour [23, 24]. The TDF consists of the following domains: knowledge, skills, social/professional identity, beliefs about capabilities, beliefs about consequences, motivation and goals, memory attention and decision processes, environmental context and resources, social influences, emotion, behavioural regulation and nature of the behaviours [24]. The interviews were analysed using directed content analysis based on the TDF [23, 24].

### Interviews

#### Subjects and setting

Recruitment for the interviews was done through eight general practices in the Northern part of the Netherlands by purposive sampling, where nurse practitioners identified and approached potential study participants. The Northern part of the Netherlands is characterized by relatively small cities, more rural areas and fewer minorities compared to other regions of the Netherlands. Most inhabitants are Caucasian. Diabetes care is organized in a similar way in all regions in the Netherlands. T2D patients were included when they used a sulfonylurea and/or insulin, experienced at least one hypoglycaemic event in the past year and were able to speak Dutch. They were excluded when they had an estimated life expectancy of less than six months or when the nurse practitioner and/or general practitioner thought they should not be approached for an interview study, because of a recently experienced serious life event. Participants were approached by their nurse practitioner to participate in the study. They were then invited by TB by phone and received a letter with information on the purpose of the study. Written informed consent was obtained and participants received a gift card as compensation (€20). Recruitment continued until saturation was achieved, which was defined as the point where no new information emerged from the interviews [25]. Data saturation was discussed between TB and SC. The interviews were conducted at the participants’ homes between January and March of 2019. Partners were allowed to be present during the interview to provide more information about, for example, medication use and experiences with severe hypoglycaemia. Field notes were taken during the interviews. The interviews were conducted by TB and SC. TB has a BSc in pharmacy and is a female pharmacy master student and SC is a male PhD candidate with an MSc in pharmacy who performed scientific interviews prior to this study. TB received instructions on how to conduct interviews and she performed a practice interview under the supervision of SC. The interviewers had no prior relationship with the patients.

#### Interview guide

A semi-structured interview guide was developed based on the domains of the TDF and known causes and self-management issues of hypoglycaemia (Additional file 1). To avoid researcher bias, open ended questions were used with additional probing questions. The interview guide was discussed among the research team and improved accordingly. The interview guide was piloted with a female patient representative, having type 1 diabetes herself. This resulted in some minor changes in the wording and the order of the questions.

Furthermore, participants completed a short questionnaire about their socio-demographic background and their lifestyle. Health literacy was assessed with the Set of Brief Screening Questions in Dutch (SBSQ-D) [26, 27]. The medication that participants used was documented by the interviewer. This medication list was confirmed with the patients’ medical records by the nurse practitioners for all but two of the participants.

#### Data analyses

Descriptive statistics were used for the patients’ characteristics. All interviews were audio recorded and transcribed verbatim using f4transkript, version 6.2.3. Transcripts were not returned to the participants. Field notes were used to enrich the transcripts with non-verbal communication of participants and contextual information. The interviews were coded using Atlas.ti, version 5.2.18. A coding frame was developed prior to the analysis of the interviews by TB and SC. It included the twelve domains of the TDF, thematic codes about the broader categories related to possible causes of hypoglycaemia and attribute codes to address important factual elements of the statements. New attribute codes were added in an iterative process to the coding frame. Both TB and SC coded all interviews, and any discrepancies between them were discussed until consensus was reached. Directed content analysis was used to analyse the data focussing on (1) self-identified causes and (2) self-management issues:

1. All quotes coded as “cause of hypoglycaemia” were extracted. This code was assigned to passages where participants talked about a possible cause of a hypoglycaemic event or where the participants mentioned that he/she did not know the cause of the event. These extractions were used to identify and categorise the self-identified cause of hypoglycaemia. Those categories were then cross-linked with “cause of hypoglycaemia” for tabulation in the results.
2. All quotes coded with domains from the TDF were cross-linked with the code “cause of hypoglycaemia” to identify self-management issues. Additionally, to identify self-management issues that were not directly linked to a cause of hypoglycaemia, all quotes coded with the TDF and without the “cause of hypoglycaemia” code were extracted and inspected for possible self-management issues related to hypoglycaemia. Quotes were selected for the manuscript in order to illustrate certain issues and to provide additional context for the reader.

### Survey

#### Subjects and setting

Participants were recruited through five community pharmacies across the Netherlands. The recruitment was done sequentially in order to facilitate age stratification. Potential participants were stratified based on age: 24% of patients < 60 years, 32% 60–70 years, 28% 70–80 years, and 16% >  = 80 years in order to have a representative sample of Dutch primary care patients with type 2 diabetes. Any differences in the age-distribution of returned questionnaires were corrected by altering the number of invitations per age-strata in following community pharmacies. The community pharmacist together with one of the researchers identified potential participants using the pharmacy information system. Inclusion criteria were: age of 40 years or older, use of a sulfonylurea and/or insulin, and able to read and write in Dutch. For the primary data analyses, patients were selected who had experienced at least one hypoglycaemic event in the past. Invitations were sent using an email with a link to the online questionnaire (Qualtrics XM). In case no email address was available a paper version was sent by mail. Informed consent was collected from all participants. For participation, patients received a gift card of €10.

#### Questionnaire

Themes from the interview study were translated to items for the questionnaire by SC and TB. These themes were related to the self-management of medication, nutrition and physical activity and to the domains of the TDF. For five of the TDF domains, items about self-management were developed in which respondents could indicate how often they experienced these self-management issues on a five-point Likert scale. For the knowledge domain, respondents were asked to indicate whether they knew how to adjust their medication in various situations in which adjustments might be necessary. Additionally, patients were asked whether or not they had experienced a hypoglycaemic event. Respondents who had at least one hypoglycaemic event in the past were then asked about self-identified causes. They were allowed to select one or more causes from a list of 18 options, that were based on the results from the interviews and literature [12–16]. All questionnaire items were discussed with PD and KT until consensus was reached about the content and phrasing. The questionnaire was piloted with four participants of the interview study. Based on the results of this pilot, a number of items were simplified and the phrasing of a number of items were improved. A translated version of the questionnaire can be found in Additional file 2. The online version of the questionnaire was created with Qualtrics Software (Qualtrics, Provo, UT).

#### Data analysis

Descriptive statistics were used to analyse the patient characteristics, the self-management issues, and the self-identified causes among respondents who had experienced at least on hypoglycaemic event. Related self-identified causes were first combined (see Additional file 2). Stacked bar charts were used to visualize the self-management issues, categorized by having experienced a hypoglycaemic event in the past. Complete case analyses were used for each item with less than 5% missing data. Descriptive statistics for all respondents, including those that had not experienced hypoglycaemic events, are summarized in Additional file 3.

### Compliance with ethical standards

A waiver was obtained by the Medical Ethics Review Board of the University Medical Center (METc UMCG) as they concluded that the study did not require approval because the study was not considered to be clinical research with human participants as meant in the Medical Research Involving Human Subjects Act.

## Results

All of the sixteen people who were invited to participate were willing to be interviewed. Data saturation occurred after sixteen interviews. The duration of the interviews ranged from 20 to 120 min. The age of the participants ranged from 59 to 84 years, ten of the participants were female, and thirteen were diagnosed with T2D more than ten years ago, eight experienced a hypoglycaemic event within one week prior to the interview (Table 1). For the survey, 208 of the 820 (25%) invited T2D patients completed the questionnaire, 83 of which (40%) had experienced at least one hypoglycaemic event in the past (Table 2). In the main analyses we report the results from the 83 respondents that had a hypoglycaemic event. On average, the respondents were 66 years old, 53% were female, and 76% were diagnosed with T2D more than ten years ago. The other respondents, who had never experienced a hypoglycaemic event, more often had a diabetes duration less than 10 years, had less diabetes related complications and used insulin less often compared to those with hypoglycaemic events (Additional file 3, Table 1).

**Table 1:** Interview participants’ characteristics (*N* = 16)

**Sex**	
Female	10
Male	6
**Age group (years)**	
< 60	2
60–70	6
70–80	4
≥ 80	4
**Level of education**	
Primary school	2
Secondary school	7
Vocational education	4
Higher education	3
**Level of health literacy**	
Low	2
Medium	0
High	14
**Use of alcohol**	
Yes	11
No	5
**Smoking**	
Yes	1
No	15
**Diabetes duration (years) **	
< 1	0
1–5	3
5–10	0
> 10	13
**Last hypoglycaemic event**	
< 1 week ago	8
1–2 weeks ago	1
2–4 weeks ago	1
> 1 month ago	6
**Glucose lowering medication**	
Insulin	4
Insulin + metformin	7
Insulin + sulfonylurea	1
Insulin + GLP-1	1
Insulin + sulfonylurea + metformin	1
Sulfonylurea	1
Sulfonylurea + metformin	1
**Glucose monitoring**	
Daily	9
Weekly	3
Rarely	3
Never	1

**Table 2:** Survey respondents’ characteristics

	**Respondents**
**Number of respondents**	83
**Age (years), mean (SD)**	66 (11)
< 60 years, n (%)	20 (24%)
60–69 years, n (%)	27 (33%)
70–79 years, n (%)	23 (28%)
≥ 80 years, n (%)	12 (14%)
Missing, n (%)	1 (1%)
**Female, n (%)**	53 (42%)
**Diabetes duration, n (%)**	
0–5 years	9 (11%)
6–10 years	11 (13%)
≥ 10 years	63 (76%)
Missing	0 (0%)
**Diabetes related complication(s)**	49 (59%)
**Body weight, n (%)**	
Underweight	0 (0%)
Healthy Weight	18 (22%)
Overweight	29 (35%)
Obese	34 (41%)
Missing	2 (2%)
**Alcohol use, n (%)**	49 (59%)
**Smoking, n (%)**	7 (8%)
**Physical activity > 30 min/day, n (%)**	
0 days	5 (6%)
1–3 days	28 (34%)
4–6 days	28 (34%)
7 days	22 (27%)
Missing	0 (0%)
**Working, n (%)**	39 (47%)
**Working irregular hours, n (%)**	13 (16%)
**Marital status/household situation (%)**	
Married/living together	55 (66%)
Living independent	27 (32%)
Missing	1 (1%)
**Education, n (%)**	
No/primary education	13 (16%)
Pre-vocational education	24 (29%)
Vocational education	15 (18%)
Pre-college/pre**-**university	11 (13%)
College/university	18 (22%)
Missing	2 (2%)
**Number of medications**	
1–5 medication(s)	32 (39%)
6–10 medications	38 (46%)
> 10 medications	11 (13%)
Missing	2 (2%)
**Insulin use**	54 (65%)
**Sulfonylurea use**	47 (57%)
**Statin use**	55 (66%)
**Antihypertensive use**	63 (76%)
**Glucose meter at home**	78 (94%)
**Severe hypoglycaemia**	15 (18%)
**Nocturnal hypoglycaemia**	31 (37%)
**Frequency hypoglycaemia**	
Daily	1 (1%)
Weekly	4 (5%)
Monthly	21 (25%)
Yearly or less	57 (69%)

### Self-management issues

Below we report the results of both the interviews and the survey categorized by the domains of the TDF that were identified in the interviews. There was overlap in the interview quotes for the domain “beliefs about capabilities” with “beliefs about consequences”, and for the domain “social/professional role and identity” with “social influences”. Therefore, the results for these domains were combined. No self-management issues were identified in the domains “behavioural regulation” and “skills".

#### Nature of behaviour

**Fig. 1:**
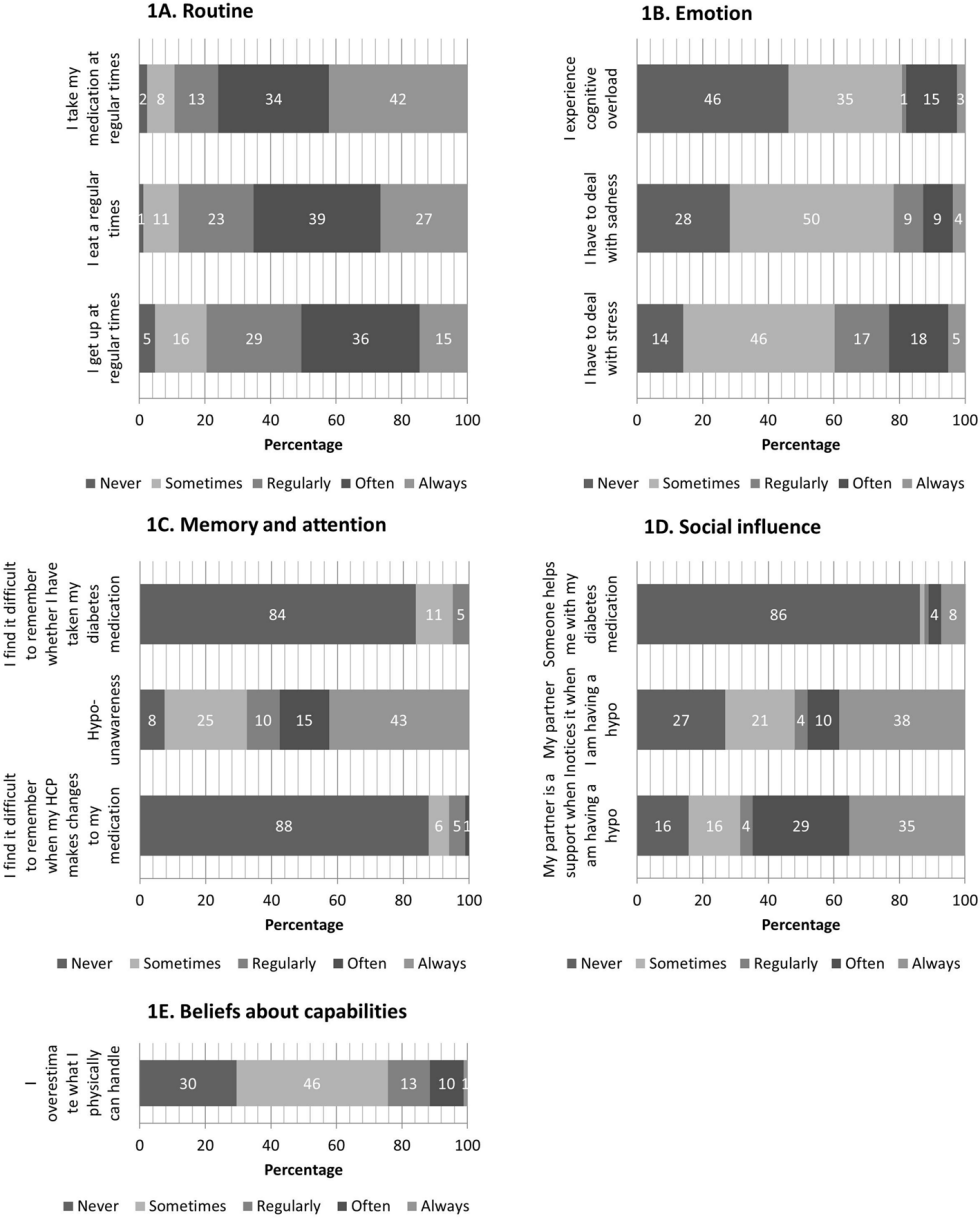
Potential self-management issues related to hypoglycaemia categorized per domain of the Theoretical Domains Framework for survey respondents with a hypoglycaemic event in the past

In the interviews, daily routine was mentioned often as an important behavioural factor for self-management and the prevention of hypoglycaemic events. Most participants had a strict daily routine and emphasized the importance of this for their self-management. A few participants reported that they had difficulties adhering to the strict routine that was needed to control their glucose levels. Some participants struggled with events that disrupted their routine which led to hypoglycaemic events (Table 3, [A]). Disruptions affected medication taking, physical activity, food intake and/or their mental state. For other participants, the rigidness of the daily routine was sometimes problematic (Table 3, [B]). In the survey, 42% of the respondents reported they always took their medication at the same time, 27% always ate at the same time and 15% always got out of bed at the same time (Fig. 1A).

**Table 3:** Quotes of participants in the interviews translated from Dutch to illustrate the self-management issues categorized by TDF domain

	**Quotes**	**TDF**
[A]	*“No, I do not have them very often* [low blood sugar]*. No, but then with my husband* [he broke his hip due to a fall] ( …) *But I just had to get used to it, to the new routine, until it was over*.*”* (female, 70–80 years old)	Nature of behaviour
[B]	*“I should have prepared the warm meal earlier, but I am used to eating lunch at 12 o’clock. And I have the food ready at 12 o’clock, and I could not make that.”* (female, 70–80 years old)	Nature of behaviour
[C]	*“I do not really know this very well yet ( …) It is not that easy for me to skip one tablet or to take one tablet extra the next day. That is my problem, because I don’t have a sufficiently regular daily schedule. I’m trying to change that.” (Female 60–70 years old)*	Knowledge/ Nature of behaviour
[D]	*“So when it is low they say: ‘you need to inject more’, ok, how much more?”* [Interviewer: “If it is low you need to inject more insulin?”] *“Yes, so that it will go up again, as it were.* [Interviewer: “Insulin lowers your sugar.”] *“Yes, exactly. So I think, I will do it my way. I get a small bottle of soda or two biscuits with jelly and then it’s all fine again.” (Female* ≥ *80 years old)*	Knowledge
[E]	*“Yes, I have had days, that I think like, ( …) I want to finish my work, my assignment, but then a colleague said like: ‘leave it for a while, we will take it over and you just sit down for a while.”* (Female < 60 years old)	Social influences and social/professional role and identity
[F]	*“I went to the gym for a while, ( …) and there I sometimes overestimated myself a little, and then I would get a hypo, because I would use a treadmill more than I could handle with my bad legs.”*	Beliefs about capabilities
[G]	*“So, I injected 4* [units] *( …) But, sometimes, it is too much and then I think that I should inject only 3 units. ( …) But then again my glucose gets close to 10 and I really want my glucose to be as low as possible.”* (Female 70–80 years old)	Motivation and goals
[H]	*“I love mashed potato stews; I allow myself this once in a while. I think I should be able to do that, otherwise life is not pleasant anymore.”* (Female 60–70 years old)	Motivation and goals

*TDF* Theoretical Domains Framework

#### Knowledge

Lacking specific knowledge to self-manage glucose levels was another important theme identified in the interviews. Participants had a good basic understanding of the relationship between their glucose levels and their medication, nutrition and physical activity. This basic understanding, however, was not always sufficient to anticipate or prevent hypoglycaemic events. Participants sometimes lacked procedural knowledge on how to address deviations from their routine, especially when medication adjustments might be necessary (Table 3, [C]). Some did not know how to adjust their insulin or they did not adjust it at all. Sometimes participants struggled to determine the right insulin dose, which led to injecting more insulin than needed. To treat the resulting low blood glucose levels they overate, which caused their blood glucose levels to rise again above the desirable threshold. This resulted in a vicious circle that they did not know how to break. Some participants reported that they experienced hypoglycaemia symptoms at relatively high glucose levels, which they managed –incorrectly- by consuming foods or drinks high in sugar. One participant reported that she was supposed to inject more insulin when the glucose level was low (Table 3, [D]). This problem of having inadequate knowledge was aggravated by the fact that she was not comfortable to ask her nurse practitioner questions, because she was afraid to appear stupid.

In the survey, the majority (94%) of respondents indicated that they had enough knowledge to use their glucose lowering medication. Many indicated that adjusting medication was not necessary when they ate or exercised more or less than usual (Table 4). Furthermore, 22% indicated they did not always know how to adjust their medication for at least one specific situation. This included 16% indicating that they did not adjust their medication in one or more of these situations because they did not know how, and 6% indicating that did adjust their medication although they did not know how. These specific situations included changes in physical activity, food intake or being ill (Table 4).

**Table 4:** Survey results of questions about knowledge on how to adjust medication in various situations which require adjustment of medication

	Yes I know how to do that	Yes, but I do not know how to do that	No, I do not know how to do that	No, I am not allowed to do so by my HCP	No that is not necessary
I adjust my medication when I exercise more than usual (*n* = 80), (%)	25.0	2.5	5.0	5.0	62.5
I adjust my medication when I exercise less than usual (*n* = 80), (%)	21.3	1.3	5.0	5.0	67.5
I adjust my medication when I eat more than usual(*n* = 82), (%)	34.2	1.2	4.9	9.8	50.0
I adjust my medication when I eat less than usual (*n* = 82), (%)	30.5	0.0	4.9	8.5	56.1
I adjust my medication based on measured glucose levels (*n* = 78), (%)	35.9	5.1	3.9	18.0	37.2
I adjust my medication when I am ill (*n* = 79), (%)	24.1	0.0	12.7	3.8	59.5
I adjust my medication when I am on a diet (*n* = 35), (%)	40.0	0.0	14.3	11.4	34.3

*HCP* Health care provider

In one or more of the situation described in the table, 22% did not know how to adjust their medication, 16% did not know how to adjust their medication in one or more of these situations and 6% did adjust their medication although they did not know how

#### Emotion

In the interviews, stress and cognitive overload were mentioned as issues interfering with managing glucose levels. Some participants reported that stress caused their glucose to rise, while others identified stress as a cause of their hypoglycaemic events. Cognitive challenging tasks, for example, administrative work on a computer, or grief could lead to hypoglycaemia. Sometimes this was due to forgetting to eat. One participant attributing some of her hypoglycaemic events to grief noted that she had to deal with grief more often due to her increasing age. Another participant lost his wife recently causing stress and grief, which resulted in poorer self-care and self-management, in turn leading to multiple hypoglycaemic events. In the survey, 40% of the respondents had to deal with stress at least regularly, 22% had to deal with sadness or grief at least regularly, and 19% experienced cognitive overload at least regularly (Fig. 1B).

#### Memory, attention and decision processes

In the interviews, issues with memory or attention were mostly stated in relation to medication taking or forgotten meals. Some participants mentioned accidentally administering more units or a double dose of insulin. When asked about accidentally using too much medication, participants often said it was possible that this happened, but they were not sure. One participant said he had once forgotten to lower the units of insulin after instructions from his nurse practitioner to do so. Another participant said he accidentally had increased his long acting insulin instead of his short acting insulin, when adjusting the dose because of a high glucose level. Impaired hypoglycaemia awareness was often mentioned in relation to severe events. Some of the participants who reported that they usually felt the warning symptoms still experienced some events with no warning signs. In the survey 16% of respondents indicated that they could not always remember whether they had already taken their medication and 12% said they had experienced difficulties in remembering dosing changes to their medication. In the survey 43% reported that they always experienced impaired hypoglycaemia awareness, whereas 7.5% never experienced impaired hypoglycaemia awareness (Fig. 1C).

#### Social influences and social/professional role and identity

In the interview, several participants mentioned that their partners played an important role in the management of the diabetes. This could be up to the extent that the partner fully managed their medication. Often partners helped the participant when they had a hypoglycaemic event, bringing them food or drinks. One participant explained that his partner noticed his hypoglycaemic events before he did. Not long after his partner passed away, he had experienced a severe hypoglycaemia, in part due to the loss of support from his partner. The work ethics of some participants led to self-management issues. Being an active person or doing a good job was important, which led to exerting themselves too much (Table 3, [E]). In the survey, 86% of the respondents reported to manage their own medication. For those with a partner, 69% of the partners at least regularly noticed hypoglycaemic events and 52% at least regularly helped when they had a hypoglycaemic event (Fig. 1D).

#### Beliefs about capabilities and consequences

In the interviews, participants mentioned issues with their ability to deal with variations in physical activity or stress. Some participants overestimated what they could do and underestimated the impact of an activity on their glucose levels (Table 3, [F]). Adapting to their diminishing physical ability was something they struggled with. One participant mentioned she felt unable to prevent the stress that led to her hypoglycaemic events. In the survey, 24% of the respondents at least regularly overestimated what they were able to handle physically (Fig. 1E).

#### Motivation and goals

In the interviews, some participants did not see hypoglycaemia as a major issue in comparison with other health-related issues. Mild events were often not considered very burdensome. Other participants, however, feared the consequences of hypoglycaemic events. One participant stated that she found it hard to navigate between too low and too high glucose levels, because she did not want hypoglycaemic events, but she also did not want her glucose to rise too much (Table 3, [G]). Some participants did not want to measure glucose too often, mainly due to the physical discomfort. For many participants, it was important that the diabetes did not take over their life, which was mostly expressed in relation to being active and dietary choices (Table 3, [H]). In the survey, 18% of the respondents had experienced a severe hypoglycaemia. Most experienced hypoglycaemic events either monthly or yearly (Table 2).

#### Environmental context and resources

In the interviews, lack of resources to use blood glucose meters and test strips were mentioned as a problem for self-management. One of the participants who used a sulfonylurea, for which blood glucose meters and test strips are not reimbursed in the Netherlands, mentioned that he sometimes used the glucose meter of his wife. Another participant stated that she was reluctant to use test strips because she felt they were too expensive. In the survey, 94.0% of the respondents had a glucose meter at home.

### Self-identified causes of hypoglycaemia

In the interviews, participants expressed that they were not always able to identify a direct cause of their hypoglycaemic events. All but one of the participants attributed at least some events to factors related to medication, nutrition, physical activity, and/or emotional burden (Table 5).

**Table 5:** Interview results: List of self-identified causes of hypoglycaemia categorised by theme and number of participants mentioning the particular theme

**Medication (6 participants)**
Accidentally overdosing medication
Forgetting adjustment made to the medication regimen
Adjusting the wrong type of insulin
Fluctuating glucose levels
**Physical activity (9 participants)**
Household chores
Sports
Physical leisure activities
Sexual activity
**Nutrition (10 participants)**
Skipped, delayed, forgotten meals
Low appetite/premature satiation
Meals low on carbohydrates
Fatty meals
Alcohol consumption
Low-carb diet
**Stress/emotion (4 participants)**
Stress
Cognitive overload
Grief

**Fig. 2:**
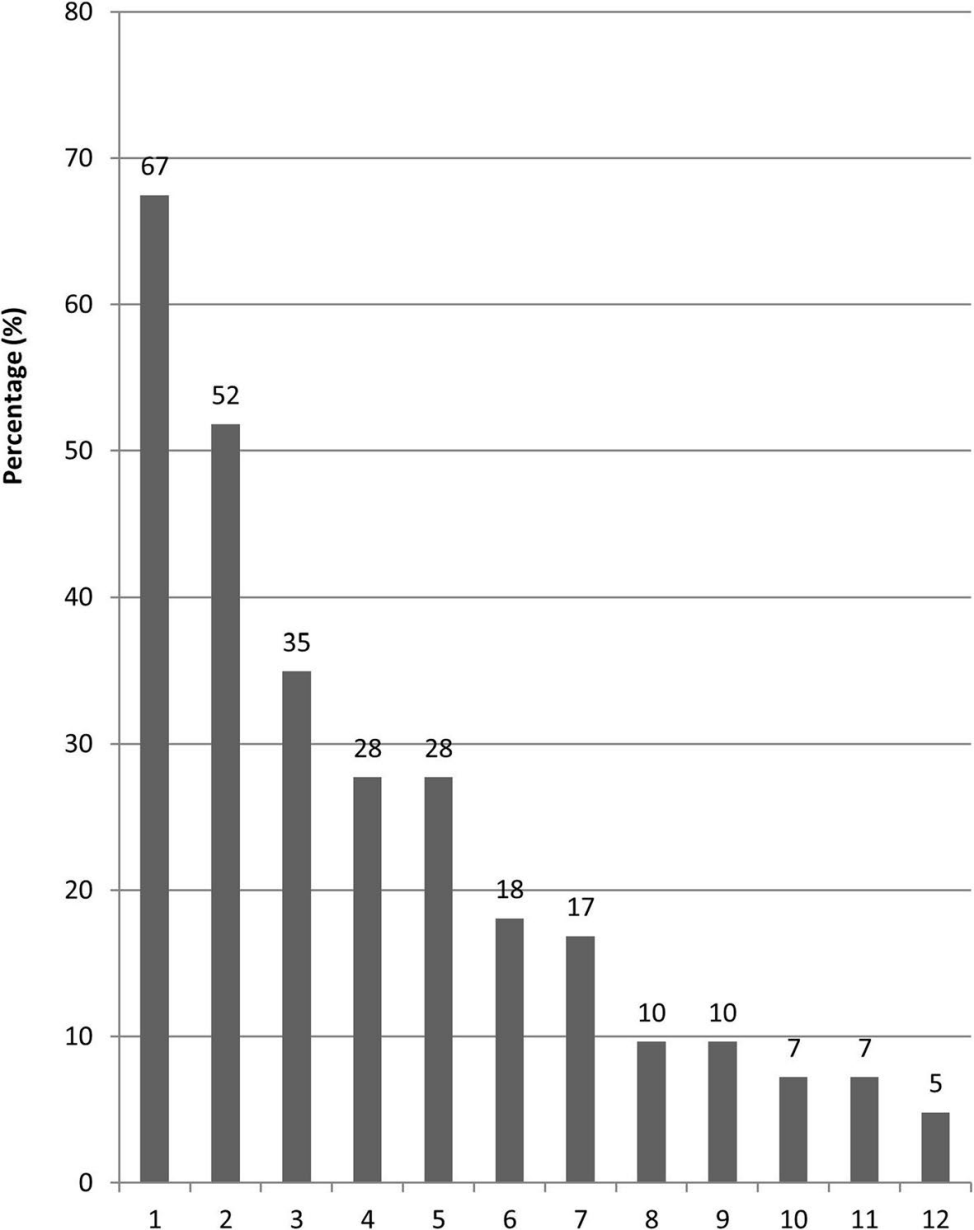
Percentage of participants reporting specific causes for their hypoglycaemia among 83 participants who had at least one hypoglycaemic event in the past. 1 - Physical activity (work/exercise); 2 - Not enough food consumed (forgotten/skipped/too small portion/late meal); 3 - Deviations from routine; 4 - Emotional burden (stress/grief/cognitive overload); 5 - Impaired hypoglycaemia awareness; 6 - No carbohydrates/sugar available to treat low blood glucose; 7 - Highly fluctuating glucose levels/poorly controlled glucose; 8 - Accidental medication overuse (e.g. injecting insulin twice or injecting more units); 9 - Hard to prevent when at work; 10 - Fat food; 11 - Lack of knowledge on how to adjust medication; 12 - Prescribed a too high dosage of sulfonylureas or insulin

In the survey, the most common self-identified causes were too much physical activity (67%), not enough food intake (52%), deviations from routines (35%) (Fig. 2). Sadness, stress or cognitive overload were reported as a cause by 28%, accidental overuse of medication was reported by 10% and lack of knowledge on how to adjust medication by 7% of the respondents.

## Discussion

### Summary

Many patients with T2D acknowledged self-management issues contributing to hypoglycaemia. Particularly, issues within the TDF domains nature of behaviour, knowledge, emotions and capabilities appeared relevant for adequate self-management and prevention of hypoglycaemic events. Although most patients seemed to have a basic knowledge about their medication and the factors that may lead to hypoglycaemia, they sometimes lacked procedural knowledge for specific situations or the ability to deal with deviations from routines or handling negative emotions such as stress and grief. The inability to correctly estimate the impact of physical activity was an issue for a quarter of the respondents. The most common self-identified causes of hypoglycaemia were issues with handling physical activity, insufficient food intake, deviations from routine, and stress and in relatively few cases also accidental overuse of medication. Some of the hypoglycaemic events were considered difficult to prevent, because the patients could not identify a cause or suffered from impaired hypoglycaemia awareness.

### Comparison with previous research

From our study, it becomes clear that the TDF domain nature of behaviour is crucial for the prevention of hypoglycaemic events. People with T2D need to manage their lifestyle and medication use to keep their glucose levels adequately controlled and prevent hypoglycaemic events. Routine behaviour plays an important role in adequate self-management. People with T2D particularly have difficulties with medication self-management when they change their daily routine [28]. Our study showed that both short-term deviations from routine behaviour as well as long-term changes in daily life could lead to problems. In acute situations, patients experienced issues with medication self-management, such as difficulties interpreting glucose levels and adjusting medication when needed. Providing instructions how to adjust insulin can reduce the frequency of hypoglycaemia in T2D patients [29]. Over time, changes in a person’s life may lead to new self-management issues. Some have to do with permanent changes in medication regimen or diet, which require adjustments that are sometimes forgotten. Others are related to loss of support or changes in physical abilities or appetite due to aging.

Diabetes-related knowledge, beliefs and skills are considered important for self-management of people with T2D [21, 30]. Our study indicates that patients have basic knowledge about the relationship between nutrition, physical activity and medication with glucose levels. They acknowledged that changes in any of these factors could cause hypoglycaemic events, and they believed to have enough knowledge to use their diabetes medication. However, some of them seem to lack the ability to translate general knowledge into adequate actions to prevent hypoglycaemic events from happening. This is in line with findings from a recent study among T2D patients with low health literacy, where most participants first expressed that they had an adequate level of medication self-management but additional questions showed that this was often insufficient [28].

Negative emotions such as stress, grief and cognitive overload can influence self-management. Stress is a factor that may have mixed effects on glucose levels. Stress can increase glucose levels but stress can also hinder self-management in diabetes patients [31, 32]. Our study confirms that many patients with T2D experience stress regularly, which may be difficult to manage by T2D patients [32]. Previous research showed that the impact of stress on self-management could be particularly problematic in diabetes patients with low self-efficacy [31].

Belief about capabilities can play an important role in how older people deal with physical activity. Especially inactive older people tend to overestimate what they can handle physically, increasing the risk of hypoglycaemia [33]. Physical activity not only increases energy expenditure but can also result in a prolonged increase in insulin sensitivity. To prevent hypoglycaemia after exercising less insulin or additional carbohydrate intake is needed [34]. Injecting less insulin is especially important to counter the increase in insulin sensitivity but few survey respondents knew how to adjust medication when they exercised more than usual.

Some specific issues with medication were identified, such as not remembering whether medication was already taken or not taking medication at regular times. Problems with memory and attention can lead to accidental overdosing of medication. In turn this can lead to a severe hypoglycaemic event but in line with other studies, patients in our study seldom acknowledged this as a cause [14, 16].

Intrinsic motivation is an important factor in engaging in self-management [35, 36]. Our interview study showed that for some patients hypoglycaemic were relatively mild and considered unimportant because other health-related issues inflicted a much higher burden on them. This low priority could prevent people with T2D from taking the necessary steps to prevent hypoglycaemic events.Most respondents in our survey did not experience very frequent or severe events.

Social influences and support can be important for self-management of chronic diseases like T2D [19]. Involving family members in self-management education has shown to have a positive effect on the management of T2D [18]. Our study also illustrated the importance of support from partners for the management and prevention of hypoglycaemic events. In the majority of the patients who have a partner, this partner often recognizes and helps dealing with hypoglycaemic events. When a T2D patient loses his or her partner the combination of stress, grief and loss of support put these patients at more risk of hypoglycaemic events.

Finally, in line with previous studies, we found that patients were not always able to identify the cause of their hypoglycaemic events [12, 13]. This leads to the perception that they cannot prevent these events. This is especially problematic since the majority suffers from impaired hypoglycaemia awareness.

## Strengths and limitations

This study is unique in investigating self-identified causes of hypoglycaemia and underlying self-management issues among people with T2D using a mixed methods design. The design allowed us to investigate in a qualitative study the behavioural related factors preceding hypoglycaemia from the perspective of patients and subsequently quantify these factors in a larger population. By using the TDF in both the development of our topic list and the coding of the interviews, we were able to get a comprehensive picture of self-management issues related to the domains that can influence behaviour and obstruct behavioural change [24]. Some limitations of this study need to be taken into account when interpreting the results. Recruitment for the interviews was done in the Northern part of the Netherlands. Although health care for people with T2D is organized similarly across the Netherlands, there are regional differences in the general population which may influence how patients experience and cope with their disease in general and with hypoglycaemia specifically. Due to the limited recruitment area and due to the exclusion of non-Dutch speaking participants we may have missed some causes of hypoglycaemia in the interviews. Due to the cross-sectional design of the survey no causal relationships can be established between hypoglycaemia and self-management issues. Furthermore, our study relies on self-report, which is inherent to interviews and surveys. Participants mentioned that they did not always know what the cause was of their hypoglycaemic events. Because we relied on self-reporting we do not know whether these events were unpredictable or that an underlying lack of knowledge made it difficult for patient to identify a cause. Furthermore, not all topics addressed in the interviews were translated to corresponding questions in the survey. For instance, where some patients expressed that their hypoglycaemic events were relatively mild and unimportant, we only quantified the frequency and severity of the hypoglycaemic events in the survey. Finally, there may be a risk of recall bias for the self-identified causes because for some of the participants the last hypoglycaemic event was relatively long ago.

## Implications for clinical practice

Our findings underline the importance of offering personalized and easy to access support to address acute problems as well as changing needs for self-management support [37]. Our findings are also useful to revise self-management programs for people with T2D. An important component of many self-management education programs is knowledge transfer [21]. Providing knowledge is an important step but may be insufficient to improve actual self-management [36]. Our study suggests that patients need hands-on practice on how to balance their medication, nutrition and physical activity when there are deviations from daily routine. Also, programs need to address how to deal with stress and how to improve self-efficacy related to managing stress, particularly in those with a lack of social support. Finally, tailored support is needed for the group of patients who suffer from hypoglycaemia in combination with poorly controlled glucose levels. Relaxing haemoglobin A1c (HbA1c) targets is not an option since they are in a vicious circle of alternating high and low glucose levels. These patients need to be trained in self-management to prevent both hypoglycaemic and hyperglycaemic events.

## Conclusion

This study provides insights in the behavioural causes of hypoglycaemia and the underlying self-management issues from the perspective of people with T2D. It underlines the importance of daily routines, having the knowledge on how to adjust medication in relation to changes in physical activity, food intake or illness, and the ability to deal with stress to prevent hypoglycaemic events.

## Guidelines and regulations

All methods were carried out in accordance with relevant guidelines and regulations.

## Supplementary Information

**Additional file 1.**

**Additional file 2.**

**Additional file 3.**

## Data Availability

The datasets generated and analysed during the current study are not publicly available as this would be in conflict with the informed consent given by the participants, but are available from the corresponding author on reasonable request.

## Abbreviations

T2D: Type 2 diabetes
TDF: Theoretical Domains Framework
SBSQ-D: Set of Brief Screening Questions in Dutch
HbA1c: Haemoglobin A1c

## References

[1] Williams SA, Pollack MF, DiBonaventura M (2011). Effects of hypoglycemia on health-related quality of life, treatment satisfaction and healthcare resource utilization in patients with type 2 diabetes mellitus. Diabetes Res Clin Pract.

[2] Wild D, von Maltzahn R, Brohan E, Christensen T, Clauson P, Gonder-Frederick L (2007). A critical review of the literature on fear of hypoglycemia in diabetes: implications for diabetes management and patient education. Patient Educ Couns.

[3] Davis S, Alonso MD (2004). Hypoglycemia as a barrier to glycemic control. J Diabetes Complications.

[4] Oyer DS. The science of hypoglycemia in patients with diabetes. Curr Diabetes Rev. 2013;9(3):195–208 Available from: ( http://www.eurekaselect.com/openurl/content.php?genre=article&issn=1573-3998&volume=9&issue=3&spage=195). 10.2174/15733998113099990059 23506375

[5] Gerstein HC, Miller ME, Genuth S, Ismail-Beigi F, Buse JB, Goff DC, et al. Long-term effects of intensive glucose lowering on cardiovascular outcomes. N Engl J Med. 2011;364(9):818–28 Available from: (http://www.nejm.org/doi/10.1056/NEJMoa1006524). 10.1056/NEJMoa1006524 PMC4083508 21366473

[6] Davis SN, Duckworth W, Emanuele N, Hayward RA, Wiitala WL, Thottapurathu L (2019). Effects of severe hypoglycemia on cardiovascular outcomes and death in the veterans affairs diabetes trial. Diabetes Care.

[7] Zinman B, Marso SP, Christiansen E, Calanna S, Rasmussen S, Buse JB (2018). Hypoglycemia, cardiovascular outcomes, and death: the LEADER experience. Diabetes Care.

[8] Silbert R, Salcido-montenegro A, Rodriguez-gutierrez R, Katabi A, Mccoy RG, Salcido-montenegro A (2018). Hypoglycemia among patients with type 2 diabetes: epidemiology, risk factors, and prevention strategies.

[9] Khunti K, Alsifri S, Aronson R, CigrovskiBerković M, Enters-Weijnen C, Forsén T (2016). Rates and predictors of hypoglycaemia in 27 585 people from 24 countries with insulin-treated type 1 and type 2 diabetes: the global HAT study. Diabetes Obes Metab.

[10] Rauh SP, Rutters F, Thorsted BL, Wolden ML, Nijpels G, Heijden Van Der AAWA (2016). Self-reported hypoglycaemia in patients with type 2 diabetes treated with insulin in the hoorn diabetes care system cohort, the Netherlands: a prospective cohort study.

[11] Gehlaut RR, Dogbey GY, Schwartz FL, Marling CR, Shubrook JH (2015). Hypoglycemia in type 2 diabetes - more common than you think: a continuous glucose monitoring study. J Diabetes Sci Technol.

[12] Murata GH, Duckworth WC, Shah JH, Wendel CS, Mohler MJ, Hoffman RM (2005). Hypoglycemia in stable, insulin-treated veterans with type 2 diabetes: a prospective study of 1662 episodes. J Diabetes Complications.

[13] Miller CD, Phillips LS, Ziemer DC, Gallina DL, Cook CB, El-Kebbi IM (2001). Hypoglycemia in patients with type 2 diabetes mellitus. Arch Intern Med.

[14] Murata GH, Duckworth WC, Hoffman RM, Wendel CS, Mohler MJ, Shah JH (2004). Hypoglycemia in type 2 diabetes: a critical review. Biomed Pharmacother.

[15] Mitchell BD, Vietri J, Zagar A, Curtis B, Reaney M (2013). Hypoglycaemic events in patients with type 2 diabetes in the United Kingdom: associations with patient-reported outcomes and self-reported HbA1c. BMC Endocr Disord.

[16] Bonds DE, Miller ME, Dudl J, Feinglos M, Ismail-Beigi F, Malozowski S, et al. Severe hypoglycemia symptoms, antecedent behaviors, immediate consequences and association with glycemia medication usage: secondary analysis of the ACCORD clinical trial data. BMC Endocr Disord. 2012;12:5 Available from: (http://www.biomedcentral.com/1472-6823/12/5%0Ahttp://ovidsp.ovid.com/ovidweb.cgi?T=JS&PAGE=reference&D=emed14&NEWS=N&AN=52039511http://www.biomedcentral.com/1472-6823/12/5%0Ahttp://ovidsp.ovid.com/ovidweb.cgi?T=JS&PAGE=reference&D=emed14&NEWS=N&AN=52039511). 10.1186/1472-6823-12-5 PMC3433360 22646230

[17] Lee JY, Wong CP, Tan CSS, Nasir NH, Lee SWH (2017). Type 2 diabetes patient’s perspective on ramadan fasting: a qualitative study. BMJ Open Diabetes Res Care.

[18] Pamungkas RA, Chamroonsawasdi K, Vatanasomboon P (2017). A systematic review: family support integrated with diabetes self-management among uncontrolled type II diabetes mellitus patients. Behav Sci (Basel).

[19] Koetsenruijter J, van Eikelenboom N, van Lieshout J, Vassilev I, Lionis C, Todorova E (2016). Social support and self-management capabilities in diabetes patients: an international observational study. Patient Educ Couns.

[20] Barlow J, Wright C, Sheasby J, Turner A, Hainsworth J (2002). Self-management approaches for people with chronic conditions: A review. Patient Educ Couns.

[21] Chatterjee S, Davies MJ, Heller S, Speight J, Snoek FJ, Khunti K (2018). Diabetes structured self-management education programmes: a narrative review and current innovations. Lancet Diabetes Endocrinol.

[22] Davies MJ, D’Alessio DA, Fradkin J, Kernan WN, Mathieu C, Mingrone G (2018). Management of hyperglycemia in type 2 diabetes, 2018. A consensus report by the American Diabetes Association (ADA) and the european association for the study of diabetes (EASD). Diabetes Care.

[23] Michie S. Making psychological theory useful for implementing evidence based practice: a consensus approach. Qual Saf Heal Care. 2005;14(1):26–33 Available from: (http://qualitysafety.bmj.com/lookup/doi/10.1136/qshc.2004.011155). 10.1136/qshc.2004.011155 PMC1743963 15692000

[24] Atkins L, Francis J, Islam R, O’Connor D, Patey A, Ivers N, et al. A guide to using the theoretical domains framework of behaviour change to investigate implementation problems. Implement Sci. 2017;12(1):77 Available from: (http://implementationscience.biomedcentral.com/articles/10.1186/s13012-017-0605-9). 10.1186/s13012-017-0605-9 PMC5480145 28637486

[25] Bowen GA (2008). Naturalistic inquiry and the saturation concept: a research note. Qual Res.

[26] Fransen MP, Van Schaik TM, Twickler TB, Essink-Bot ML (2011). Applicability of internationally available health literacy measures in the Netherlands. J Health Commun.

[27] Chew LD, Bradley KA, Boyko EJ (2004). Brief Questions to Identify Patients With Inadequate Health Literacy. Fam Med.

[28] Visscher BB, Steunenberg B, Heerdink ER, Rademakers J (2020). Medication self-management support for people with diabetes and low health literacy: a needs assessment. PLoS One.

[29] Yong YM, Shin KM, Lee KM, Cho JY, Ko SH, Yoon MH (2015). Intensive individualized reinforcement education is important for the prevention of hypoglycemia in patients with type 2 diabetes. Diabetes Metab J.

[30] Van Der Heide I, Uiters E, Rademakers J, Struijs JN, Schuit AJ, Baan CA (2014). Associations among health literacy, diabetes knowledge, and self-management behavior in adults with diabetes: results of a Dutch cross-sectional study. J Health Commun.

[31] Guo J, Yang J, Wiley J, Ou X, Zhou Z, Whittemore R (2019). Perceived stress and self-efficacy are associated with diabetes self-management among adolescents with type 1 diabetes: A moderated mediation analysis. J Adv Nurs.

[32] Adu MD, Malabu UH, Malau-Aduli AEO, Malau-Aduli BS (2019). Enablers and barriers to effective diabetes self-management: A multi-national investigation. PLoS One.

[33] McAuley E, Mailey EL, Mullen SP, Szabo AN, Wójcicki TR, White SM (2011). Growth trajectories of exercise self-efficacy in older adults: influence of measures and initial status. Heal Psychol.

[34] Colberg SR, Sigal RJ, Yardley JE, Riddell MC, Dunstan DW, Dempsey PC (2016). Physical activity/exercise and diabetes: a position statement of the American Diabetes Association. Diabetes Care.

[35] Murphy K, Casey D, Dinneen S, Lawton J, Brown F (2011). Participants’ perceptions of the factors that influence Diabetes Self-Management Following a Structured Education (DAFNE) programme. J Clin Nurs.

[36] Ahola AJ, Groop PH (2013). Barriers to self-management of diabetes. Diabet Med.

[37] Johnson EL, Frias JP, Trujillo JM (2018). Anticipatory guidance in type 2 diabetes to improve disease management; next steps after basal insulin. Postgrad Med.

