# Supplementary material for: Type 2 diabetes patients’ views on prevention of hypoglycaemia – a mixed methods study investigating self-management issues and self-identified causes of hypoglycaemia
Source: BMC Fam Pract. 2021 Jun 14;22:114. doi: 10.1186/s12875-021-01466-0 (PMC8210634; doi:10.1186/s12875-021-01466-0)
Supplement: Supplementary file 1 — Additional file 1. [file 12875_2021_1466_MOESM1_ESM.docx]

# Type 2 diabetes patients’ views on prevention of hypoglycaemia

# – a mixed methods study investigating self-management issues and self-identified causes of hypoglycaemia

Stijn Crutzen^1^, Tessa van den Born-Bondt^1^, Petra Denig^1^ and Katja Taxis^2^

1 Department of Clinical Pharmacy and Pharmacology, University Medical Centre Groningen, University of Groningen, Groningen, Netherlands

2 Unit of PharmacoTherapy, Epidemiology and Economics, Groningen Research Institute of Pharmacy, University of Groningen, Groningen, The Netherlands

**Additional file I**

**Interview guide**

**Prior to the interview:**

Short introduction:

Before we begin with the interview I will shortly explain what the aim is of the interview. Do you know what I mean when I talk about a hypo or hypoglycaemia?

We want to know what kind of problems you might experience concerning your hypo’s. Among other thing we want to explore the role of your diabetes medication on your hypo’s. Off course other factors could also result in getting a hypo, during the interview you can talk about this.

During the interview I will shortly introduce each part of the interview. The interview will be recorded, in order to not miss anything you say during the interview. Additionally I will make notes during the interview on the thing you talk about during the interview.

1) Do you have any questions at this point?

**Start interview:**

Then I would like to start with the interview. To begin with I would like to know a bit more about your life with diabetes. I would like to know how you handle your medication use, your diet, your exercise and other things that are important because of your diabetes.

2) Could you tell me how a normal day looks like for you concerning your diabetes? You can think about things like medication use, diet and exercise.

Probing questions:

*a. How do you monitor your blood sugar?*

*b. What do you do with the measurements?*

*c. How active are you? (bicycling, driving a car, sports, household chores)*

*d. What are your eating habits?*

*e. What are your living arrangement? (partner, single, children, care taker, home care?)*

*f. Are all days and weeks the same?*

*You just talked about your daily life how this is affected by your diabetes. You were selected for this study by your nurse practitioner, because you experiences hypo’s occasionally. That is why in the next part of the interview we want to talk about how often you get hypo’s, the symptoms you experience and what you do when you get a hypo.*

3) When was your last hypo? Could you tell something about?

4) Did you feel any warning signs when you got this hypo?

*a. Do you always get warning signs?*

5) What were the signs you got?

*a. What is the first thing you notice?*

*b. Is this always the same? Are there any other symptoms you get?*

*c. Which other symptoms do you know that are associated with hypo’s?*

6) What did you do when you got your last hypo?

*a. Is this always the same?*

*b. Do you think this is easy or hard to do?*

*c. What stops you to do this?*

7) Have you ever had a severe hypo? (if needed, explain what is considered a severe hypo)

*a. Can you tell something about this?*

*b. What did you noticed?*

*c. What did you do?*

*d. What were the differences with a mild hypo?*

8) What consequences do you notice after a hypo?

*a. Are there things you do not do anymore because of your hypo’s?*

9) How burdensome are your hypo’s?

The next part of the interview will be about the causes of your hypo’s. About the thing that happen before you get a hypo.

10) You just talked about your last hypo. What do you think was the cause of this hypo

*a. What do you think was the cause of the severe hypo?*

*b. Is this always the cause of your hypo’s?*

11) Why do you think you get hypo’s?

*a. Does it have something to do with the things you eat?*

*b. Does it have something to do with the how physically active you are?*

*c. Does it have something to do with how you use your medication?*

*d. Does it have something to do with being ill?*

12) Could you tell something about the circumstances before a hypo that influence getting a hypo?

*a. How do you feel before a hypo?*

You talk about the possible causes of your hypo’s. For this study we are especially interested in the use of medication. That is why the next part of the interview will be about your medication. This will be about the medication you use for your diabetes as well as the medication you might be using for other reasons.

13)  *Which medicines do you use for your diabetes*?

*a. What do you think about the medication you use?*

*b. How do you use these medication?*

*c. Are you always able to do this? Why not?*

*d. What do you do when you forget to take your medication?*

*e. Do your medicines work?*

*f. Do you have side effects?*

*g. Do you experience any problems with your medication?*

*h. Did you use other medication before? How did you like that?*

14) Most likely you have received a lot of information about your diabetes and about the medication for your diabetes from your general practitioner, community pharmacist and your nurse practitioner. What do you think about the information you received about your diabetes medication?

15) Do you use any other medication?

*a. What is the reason for taking this medication?*

16) Do you have any other diseases for which you do not use any medication?

17) Do you think that these diseases influence your sugar levels?

*a. If yes: How?*

*b. Do you alter your non-diabetes medication sometimes because of your diabetes?*

18) What do you think would happen if you were to take less medication to lower your sugar level?

In the next part I would like to talk about the influence of your social surroundings on your diabetes and on your hypo’s.

19) Do you talk about your diabetes with people in your surrounding like family, friends, your partner or care taker?

*a. Yes: What do you talk about? No: why not?*

*b. Do you talk about hypo’s?*

20) Are there any other people who help you with your diabetes or your medication?

*a. Do they also help with hypo’s?*

*b. What do they do to help?*

21) What do people think about your diabetes en your hypo’s?

*a. How does this affect you?*

Optional:

We talked about the causes of the hypo you experience and about your medication use. At the beginning of the interview you completed a short questionnaire with some questions about your daily life. I would like to talk about some of these questions with you.

22) How often do you smoke?

*a. What is the influence of this on your hypo’s?*

23) How much alcohol do you drink per week?

*a. What is the influence of this on your hypo’s?*

We are almost at the end of the interview.

24) Are there any topics that we have not discussed yet that might help me to understand the topic better?
