# Supplementary material for: Type 2 diabetes patients’ views on prevention of hypoglycaemia – a mixed methods study investigating self-management issues and self-identified causes of hypoglycaemia
Source: BMC Fam Pract. 2021 Jun 14;22:114. doi: 10.1186/s12875-021-01466-0 (PMC8210634; doi:10.1186/s12875-021-01466-0)
Supplement: Supplementary file 2 — Additional file 2. [file 12875_2021_1466_MOESM2_ESM.docx]

# Type 2 diabetes patients’ views on prevention of hypoglycaemia

# – a mixed methods study investigating self-management issues and self-identified causes of hypoglycaemia

Stijn Crutzen^1^, Tessa van den Born-Bondt^1^, Petra Denig^1^ and Katja Taxis^2^

1 Department of Clinical Pharmacy and Pharmacology, University Medical Centre Groningen, University of Groningen, Groningen, Netherlands

2 Unit of PharmacoTherapy, Epidemiology and Economics, Groningen Research Institute of Pharmacy, University of Groningen, Groningen, The Netherlands

**Additional file II. Translated questionnaire**

What is your gender?

- Male
- Female
- Different, namely:____________

What is your age?______________

How tall are you? (in centimeters)______________

What is your marital state?

- Single (never married)
- Married/living together
- Divorced
- Widow/widower

What is your highest level of education (including diploma)?

- No/primary education
- Pre-vocational education
- Vocational education
- Pre-college/pre**-**university
- College/university

Do you work and / or do you volunteer work?

- Yes, I have regular working hours
- Yes, I have irregular working hours
- No

Do you smoke?

- Yes
- No

Do you drink alcohol?

- Never
- Every month or less
- 2-4 times a month
- 2-3 times in a week
- 4 times or more in a week

If yes, do you drink strong liquor?

- Never
- Every month or less
- 2-4 times a month
- 2-3 times in a week
- 4 times or more in a week

How many days in a week do you exercise 30 minutes or more (including outdoor walking)?0

- 1
- 2
- 3
- 4
- 5
- 6
- 7

How long have you had diabetes?

- Less than 1 year
- 1 to 5 years
- 6-10 years
- 10 years or longer

Have you developed one or more of the following problems due to your diabetes? You can choose multiple answers.

- Nerve damage due to diabetes, complaints of which are often tingling, a burning / nagging pain or a numbness in your feet or lower legs. (Neuropathy)
- Kidney damage due to diabetes (Nephropathy)
- Eye damage due to diabetes (Retinopathy)
- Other problems, namely:____________________________________
- No problems

How many different types of medicines do you use in total? (Including insulin)

- 1-5 medicines
- 6-10 medicines
- More than 10 medicines

**Hypoglycemia:** The amount of sugar in your blood changes throughout the day. Low blood sugar is called hypoglycaemia (hypo). The following questions will be about hypos.

Have you ever had a hypo

- Yes
- No

You only need to answer this question if you have ever had a hypo.

How often do you have a hypo?

- Daily
- Several times a week, but not every day
- Several times a month but not every week
- A few times a year or less
- Never

You only need to answer this question if you have ever had a hypo.

Have you ever had a really bad hypo? This means that you had to be helped by someone else because your blood sugar was too low to treat yourself.

- Yes
- No

You only need to answer this question if you have ever had a severe hypo.

How often have you had a really bad hypo? This means that you had to be helped by someone else because your blood sugar was too low to treat yourself.

- 0 times
- 1 time
- 2-5 times
- More than 5 times

You only need to answer this question if you have ever had a hypo.

Have you ever had a hypo at night?

- Yes
- No

Do you have your own glucose meter at home

- Yes
- No, but my partner does and I use it sometimes
- No

How often did the following apply to your life in the past few months?

|  | Never | Sometimes | Regularly | Often | Always |
| --- | --- | --- | --- | --- | --- |
| I sleep more than 10 hours a night. |  |  |  |  |  |
| I get out of bed at the same time |  |  |  |  |  |
| I take my medication at regular intervals. |  |  |  |  |  |
| I eat at fixed times. |  |  |  |  |  |
| I have to deal with stress |  |  |  |  |  |
| I have to deal with sadness |  |  |  |  |  |
| I feel mentally exhausted |  |  |  |  |  |
| I find it difficult to remember if I have taken / injected my diabetes medications |  |  |  |  |  |
| I find it difficult to remembering how much to medication to use when my healthcare provider has adjusted the amount of diabetes medications. |  |  |  |  |  |
| I overestimate what I can physically handle. |  |  |  |  |  |

You only need to answer this question if you have ever had a hypo.

How often did the following apply to your life in the past few months?

|  | Never | Sometimes | Regularly | Often | Always |
| --- | --- | --- | --- | --- | --- |
| I notice when I get a hypo. |  |  |  |  |  |
| My partner is an important support when I am having a hypo |  |  |  |  |  |
| My partner notices it when I am having a hypo |  |  |  |  |  |

I adjust my diabetes medication based on my blood glucose levels

- Yes, because I know how to do that.
- Yes, but I don't really know how to do that.
- Sometimes, because I know how to do that.
- Sometimes, but I don't really know how to do that.
- No, because I don't know how to do that.
- No, because I am not allowed to do so by my care provider.
- No, because that is not necessary.

*responses on the sometimes anwsers were added to the yes responses

I adjust my diabetes medications if I eat more than usual

- Yes, because I know how to do that.
- Yes, but I don't really know how to do that.
- Sometimes, because I know how to do that.
- Sometimes, but I don't really know how to do that.
- No, because I don't know how to do that.
- No, because I am not allowed to do so by my care provider.
- No, because that is not necessary.

*responses on the sometimes anwsers were added to the yes responses

I adjust my diabetes medications if I eat less than usual

- Yes, because I know how to do that.
- Yes, but I don't really know how to do that.
- Sometimes, because I know how to do that.
- Sometimes, but I don't really know how to do that.
- No, because I don't know how to do that.
- No, because I am not allowed to do so by my care provider.
- No, because that is not necessary.

*responses on the sometimes anwsers were added to the yes responses

I adjust my diabetes medications if I exercise more than usual.

- Yes, because I know how to do that.
- Yes, but I don't really know how to do that.
- Sometimes, because I know how to do that.
- Sometimes, but I don't really know how to do that.
- No, because I don't know how to do that.
- No, because I am not allowed to do so by my care provider.
- No, because that is not necessary.

*responses on the sometimes anwsers were added to the yes responses

I adjust my diabetes medications if I exercise less than usual.

- Yes, because I know how to do that.
- Yes, but I don't really know how to do that.
- Sometimes, because I know how to do that.
- Sometimes, but I don't really know how to do that.
- No, because I don't know how to do that.
- No, because I am not allowed to do so by my care provider.
- No, because that is not necessary.

*responses on the sometimes anwsers were added to the yes responses

I adjust my diabetes medications when I'm on a diet. For example when following a carbohydrate-restricted diet.

- Yes, because I know how to do that.
- Yes, but I don't really know how to do that.
- Sometimes, because I know how to do that.
- Sometimes, but I don't really know how to do that.
- No, because I don't know how to do that.
- No, because I am not allowed to do so by my care provider.
- No, because that is not necessary.

*responses on the sometimes anwsers were added to the yes responses

I adjust my diabetes medications when I am ill.

- Yes, because I know how to do that.
- Yes, but I don't really know how to do that.
- Sometimes, because I know how to do that.
- Sometimes, but I don't really know how to do that.
- No, because I don't know how to do that.
- No, because I am not allowed to do so by my care provider.
- No, because that is not necessary.

*responses on the sometimes anwsers were added to the yes responses

I have sufficient knowledge about my diabetes medications to use them properly

- Yes
- No

**You only need to answer this question if you have ever had a hypo.**

What do you think will give you a hypo? Read all options carefully.

*You can choose several options. Choose the options that best suit you.*

- Because I do not notice that I am about to get a hypo.
- Because sometimes I accidentally inject my insulin twice.
- Because I sometimes accidentally inject too much insulin
- When I have to think a lot about my activities
- When I deviate from my daily routine
- When I exercise more than normal (household tasks, sports, recreational cycling or walking, etc.)
- Because I do pysically demanding work
- When I am sad
- When I am stressed
- Because my glucose is difficult to control.
- When I eat fatty foods (e.g. French fries).
- When I am so busy I forget to eat
- When I do not know how to adjust my medication
- When I do not have the nerve to communicate at work that I am getting a hypo.
- When I am working, I find it harder to avoid a hypo.
- Because I accidentally took too many tablets for my diabetes.
- Because I have to take too much medication for my diabetes from my healthcare provider.
- Because I did not have fast acting sugar / cookies or anything with me to treat low blood sugar.
- Different, namely:
