# Supplementary material for: Type 2 diabetes patients’ views on prevention of hypoglycaemia – a mixed methods study investigating self-management issues and self-identified causes of hypoglycaemia
Source: BMC Fam Pract. 2021 Jun 14;22:114. doi: 10.1186/s12875-021-01466-0 (PMC8210634; doi:10.1186/s12875-021-01466-0)

# Type 2 diabetes patients’ views on prevention of hypoglycaemia

# – a mixed methods study investigating self-management issues and self-identified causes of hypoglycaemia

Stijn Crutzen^1^, Tessa van den Born-Bondt^1^, Petra Denig^1^ and Katja Taxis^2^

1 Department of Clinical Pharmacy and Pharmacology, University Medical Centre Groningen, University of Groningen, Groningen, Netherlands

2 Unit of PharmacoTherapy, Epidemiology and Economics, Groningen Research Institute of Pharmacy, University of Groningen, Groningen, The Netherlands

**Additional file III. Results of the survey comparing respondents with and without hypoglycaemic events in the past**

Table 1. Survey respondents’ characteristics comparing respondent with and without a hypoglycaemic event in the past

|  | **Total** | **Hypoglycaemic event in the past** | **No hypoglycaemic event in the past** |
| --- | --- | --- | --- |
| **Number of respondents** | 208 | 83 (40%) | 125 (60%) |
| **Age (years), mean (SD)** | 68 (11) | 66 (11) | 69 (10) |
| <60 years, n (%) | 41 (20%) | 20 (24%) | 21 (17%) |
| 60-69 years, n (%) | 71 (34%) | 27 (33%) | 44 (35%) |
| 70-79 years, n (%) | 61 (29%) | 23(28%) | 38 (30%) |
| ≥80 years, n (%) | 34 (16%) | 12 (14%) | 22 (18%) |
| Missing, n (%) | 1 (1%) | 1 (1%) | 0 (0%) |
| **Female, n (%)** | 92 (44%) | 53 (42%) | 39 (48%) |
| **Diabetes duration, n (%)** |  |  |  |
| 0-5 years | 32 (15%) | 9 (11%) | 23 (19%) |
| 6-10 years | 50 (24%) | 11 11 (13%) | 39 (31%) |
| ≥10 years | 125 (60%) | 63 (76%) | 62 (50%) |
| Missing | 1 (1%) | 0 (0%) | 1(1%) |
| **Diabetes related complication(s)** | 93 (45%) | 49 (59%) | 44 (35%) |
| **Body weight, n (%)** |  |  |  |
| Underweight | 0 (%) | 0 (0%) | 0 (0%) |
| Healthy Weight | 35 (17%) | 18 (22%) | 17 (14%) |
| Overweight | 89 (43%) | 29 (35%) | 60 (48%) |
| Obese | 81 (39%) | 34 (41%) | 47 (38%) |
| Missing | 3 (1%) | 2 (2%) | 1 (1%) |
| **Alcohol use, n (%)** | 126 (61%) | 49 (59%) | 77 (62%) |
| **Smoking, n (%)** | 20 (10%) | 7 (8%) | 13 (10%) |
| **Physical activity > 30 min/day, n (%)** |  |  |  |
| 0 days | 11 (5%) | 5 (6%) | 6 (5%) |
| 1-3 days | 72 (35%) | 28 (34%) | 44 (35%) |
| 4-6 days | 66 (32%) | 28 (34%) | 38 (30%) |
| 7 days | 58 (28%) | 22 (27%) | 36 (29%) |
| Missing | 1 (0.5%) | 0 (0%) | 1 (1%) |
| **Working, n (%)** | 89 (43%) | 39 (47%) | 50 (40%) |
| **Working irregular hours, n (%)** | 32 (15%) | 13 (16%) | 19 (15%) |
| **Marital status/household situation (%)** |  |  |  |
| Married/living together | 136 (65%) | 55 (66%) | 81 (65%) |
| Living independent | 71 (34%) | 27 (32%) | 44 (35%) |
| Missing | 1 (1%) | 1 (1%) | 0 (0%) |
| **Education, n (%)** |  |  |  |
| No/primary education | 26 (13%) | 13 (16%) | 13 (10%) |
| Pre-vocational education | 71 (35%) | 24 (29%) | 47 (38%) |
| Vocational education | 37 (18%) | 15 (18%) | 22 (18%) |
| Pre-college/pre**-**university | 28 (14%) | 11 (13%) | 17 (14%) |
| College/university | 42 (21%) | 18 (22%) | 24 (19%) |
| Missing | 4 (2%) | 2 (2%) | 2 (2%) |
| **Number of medications** |  |  |  |
| 1-5 medication(s) | 94 (45%) | 32 (39%) | 62 (49%) |
| 6-10 medications | 90 (43%) | 38 (46%) | 52 (42%) |
| >10 medications | 21 (10%) | 11 (13%) | 10 (8%) |
| Missing | 3 (1%) | 2 (2%) | 1 (1%) |
| **Insulin use** | 87 (42%) | 54 (65%) | 33 (26%) |
| **Sulfonylurea use** | 159 (76%) | 47 (57%) | 112 (90%) |
| **Statin use** | 134 (64%) | 55 (66%) | 79 (63%) |
| **Antihypertensive use** | 145 (70%) | 63 (76%) | 82 (66%) |
| **Glucose meter at home** | 153 (74%) | 78 (94%) | 75 (60%) |
| **Severe hypoglycaemia** | 15 (7%) | 15 (18%) | 0 (0%) |
| **Nocturnal hypoglycaemia** | 31 (15%) | 31 (37%) | 0 (0%) |
| **Frequency hypoglycaemia** |  |  |  |
| Daily | 1 (1%) | 1 (1%) | 0 (%) |
| Weekly | 4 (2%) | 4 (5%) | 0 (%) |
| Monthly | 21 (10% ) | 21 (25%) | 0 (%) |
| Yearly or less | 57 (27%) | 57 (69%) | 0 (%) |

Table 2. Survey results of questions about knowledge on how to adjust medication in various situations which require adjustment of medication comparing respondent with and without a hypoglycaemic event in the past

|  |  | Yes I know how to do that | Yes, but I do not know how to do that | No, I do not know how to do that | No, I am not allowed to do so by my HCP | No that is not necessary |
| --- | --- | --- | --- | --- | --- | --- |
| **I adjust my medication when I exercise more than usual** | No hypo (n=118), (%) | 6.8 | 0.0 | 11.9 | 0.9 | 80.5 |
|  | Hypo(n=80), (%) | 25.0 | 2.5 | 5.0 | 5.0 | 62.5 |
|  | Total(n=198) (%) | 14.0 | 1.0 | 9.0 | 3.0 | 73.0 |
| **I adjust my medication when I exercise less than usual** | No hypo(n=117), (%) | 5.1 | 0.0 | 6.8 | 1.7 | 86.3 |
|  | Hypo(n=80), (%) | 21.3 | 1.3 | 5.0 | 5.0 | 67.5 |
|  | Total(n=197), (%) | 11.7 | 0.5 | 6.1 | 3.1 | 78.7 |
| **I adjust my medication when I eat more than usual** | No hypo(n=121), (%) | 6.6 | 0.8 | 9.9 | 9.1 | 73.6 |
|  | Hypo(n=82), (%) | 34.2 | 1.2 | 4.9 | 9.8 | 50.0 |
|  | Total(n=203), (%) | 18.0 | 1.0 | 8.0 | 9.0 | 64.0 |
| **I adjust my medication when I eat less than usual** | No hypo(n=121), (%) | 6.6 | 1.7 | 9.1 | 8.3 | 74.4 |
|  | Hypo(n=82), (%) | 30.5 | 0.0 | 4.9 | 8.5 | 56.1 |
|  | Total(n=203), (%) | 16.2 | 1.0 | 7.4 | 8.4 | 67.0 |
| **I adjust my medication based on measured glucose levels** | No hypo(n=73), (%) | 17.8 | 0.0 | 11.0 | 16.4 | 54.8 |
|  | Hypo(n=78), (%) | 35.9 | 5.1 | 3.9 | 18.0 | 37.2 |
|  | Total(n=151), (%) | 27.0 | 3.0 | 7.0 | 17.0 | 46.0 |
| **I adjust my medication when I am ill** | No hypo(n=118), (%) | 8.5 | 2.5 | 11.0 | 4.2 | 73.7 |
|  | Hypo(n=79), (%) | 24.1 | 0.0 | 12.7 | 3.8 | 59.5 |
|  | Total(n=197), (%) | 14.7 | 1.5 | 11.7 | 4.1 | 68.0 |
| **I adjust my medication when I am on a diet** | No hypo(n=45), (%) | 17.8 | 2.2 | 22.2 | 8.9 | 48.9 |
|  | Hypo(n=35), (%) | 40.0 | 0.0 | 14.3 | 11.4 | 34.3 |
|  | Total(n=80), (%) | 27.5 | 1.3 | 18.8 | 10.0 | 42.5 |

HCP: Health care provider

Figure 1. Potential self-management issues related to hypoglycaemia categorized per domain of the Theoretical Domains Framework comparing survey respondents with and without a hypoglycaemic event in the past
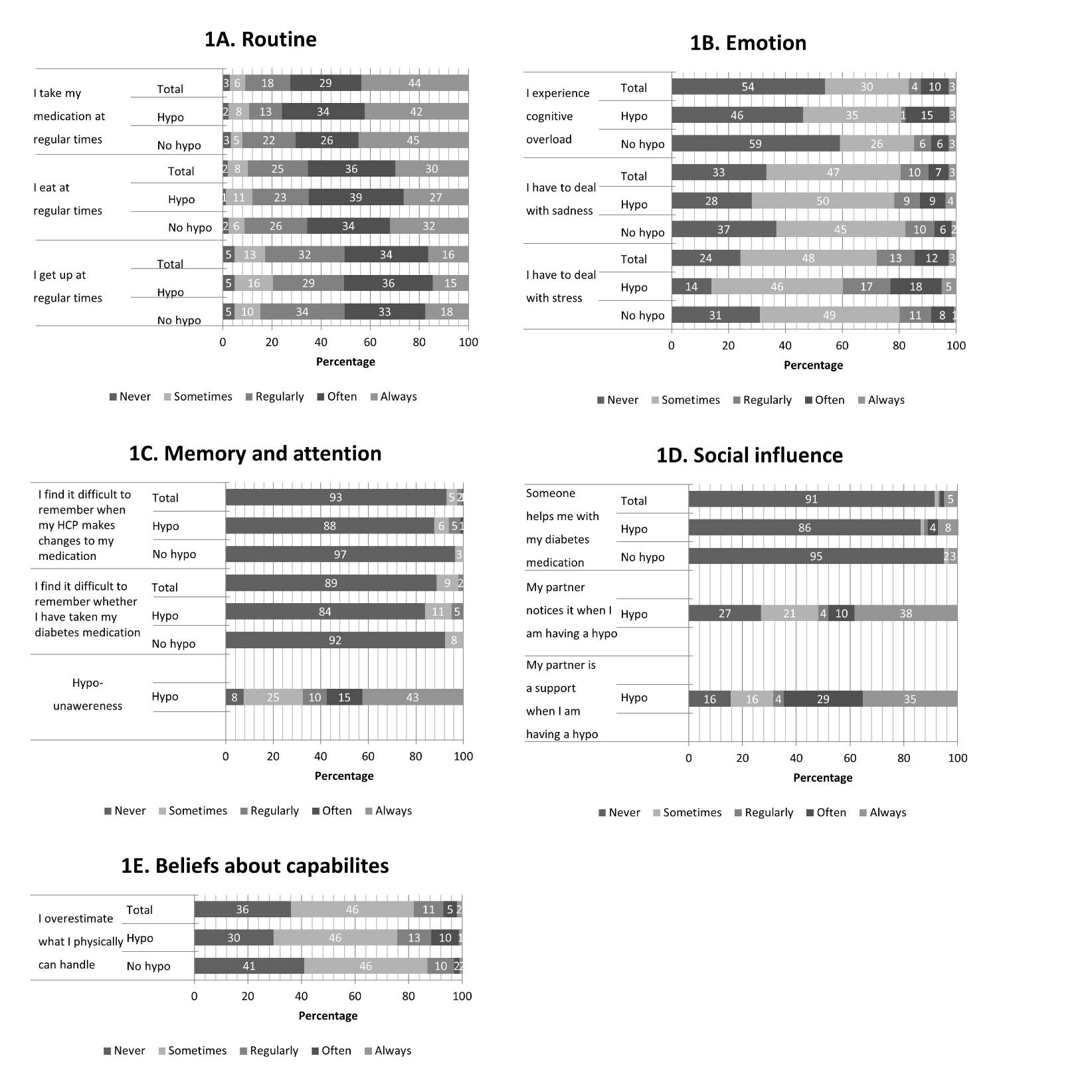

Supplement: Supplementary file 3 — Additional file 3. [file 12875_2021_1466_MOESM3_ESM.docx]
